# Physical activity, screen time and obesity status in a nationally representative sample of Maltese youth with international comparisons

**DOI:** 10.1186/1471-2458-14-664

**Published:** 2014-06-28

**Authors:** Andrew Decelis, Russell Jago, Kenneth R Fox

**Affiliations:** 1 Institute for Physical Education and Sport, University Sports Complex, University of Malta, Msida, MSD2080, Malta; 2 Centre for Exercise, Nutrition and Health Sciences, School for Policy Studies, University of Bristol, 8, Priory Road, Bristol BS8 1TZ, UK

**Keywords:** Obesity, Overweight, Physical activity, Screen time, Sedentary time, Measurement, Accelerometry

## Abstract

**Background:**

There is some evidence that physical activity (PA), sedentary time and screen time (ST) are associated with childhood obesity, but research is inconclusive and studies are mainly based on self-reported data. The literature is dominated by data from North American countries and there is a shortage of objective data from Malta which has one of the highest prevalences of childhood obesity in the world. The aims of this study were to assess the PA levels and ST patterns of Maltese boys and girls and how they compared with children in other countries while also examining differences in PA and ST by weight status.

**Methods:**

A nationally representative sample of 1126 Maltese boys and girls aged 10–11 years, of which 811 provided complete data. Physical activity was assessed using accelerometry, and ST by questionnaire. Body mass index (BMI) was computed from measured height and weight.

**Results:**

Only 39% of boys and 10% of girls met the recommendation of one hour of daily MVPA. Comparison with international data indicated that mean MVPA (58.1 min for boys; 41.7 min for girls) was higher than in North America and Australia, but lower than in England. Girls were less active than boys at all measured times and spent less time in ST. A quarter of the children exceeded guidelines of two hours of TV on weekends, and double the amount on weekdays. Obese children were less active than normal weight children on weekdays and on weekends, reaching significance during the period after school, and they spent more time in ST than their normal weight counterparts.

**Conclusions:**

A low percentage of Maltese 10–11 year olds, particularly girls, reached the recommended levels of daily MVPA and spent large amounts of time engaged in screen time. Obese children were less active than non-obese children. As children spend most of their waking time at school and that activity during this time is less than one third of the daily requirements, aiming to increase MVPA at school for all Maltese children is likely to be an important strategy to promote MVPA. Targeting less active and obese children is important.

## Background

Physical activity is associated with improved physical and mental well-being among children and adolescents [1]. The percentage of children meeting the current PA guidelines of at least 60 minutes of moderate to vigorous physical activity (MVPA) a day [1] is low in many European countries, the United States and Canada [2,3]. A large percentage of children and adolescents spend considerable amounts of time being sedentary [3], and exceed the recommendation of not more than two hours of screen time per day [4].

There is inconclusive evidence of the association between physical activity and weight status among young people [5]. A recent review of cross-sectional studies conducted over the last ten years reported a negative relationship between PA and child weight status in some studies and no association in others. Sedentary behaviours were positively associated with higher weight status. However, results were inconsistent for total sedentary time and different types of screen time and effects varied for boys and girls [6,7]. A number of studies have reported that screen time is associated with body mass among children [8,9]. Thus, more information on the link between body mass and both physical activity and sedentary time is needed.

It is important to highlight that most of the studies that have assessed associations between physical activity, sedentary time and body mass have used self-reported measures of physical activity and screen time which are subject to response and recall bias [10]. Self-reported height and weight have been reported as underestimating overweight prevalence in comparison to measured height and weight [11]. This highlights the need for objective measurements of PA, sedentary time and weight status [6] as they provide a more precise estimate of these variables.

Current research is also limited by the location as most studies have been undertaken in North America, in Portugal and in the UK. As such, research is needed on physical activity and weight status using objective measures in other countries, particularly where there is a high prevalence of obesity.

Studies using self-report measures have suggested that Maltese children have the second highest prevalence of overweight and obesity at age 11 after USA [12]. Even though the problem of obesity in Maltese children and adults seems to be severe, data on lifestyle factors, including physical activity, sedentary behaviours and their association with obesity is limited. Furthermore, only one study has been published where activity and height and weight have been measured objectively and it has been limited to 11–12 year olds [13]. This was a pilot study by the authors and indicated very low physical activity levels, and high sedentary time, screen time and prevalence of obesity. However, the sample size was small and not nationally representative.

In the present study, we systematically selected a nationally representative sample of Maltese 10–11 year boys and girls and assessed through objective measures, PA, sedentary behaviour and screen time in different weight status categories. The resulting data were used to address the following research questions: 1) What are their physical activity levels? 2) What are their screen time (ST) patterns? 3) How do their physical activity and screen time levels compare to other EU and non EU countries? 4) How do their physical activity and screen time patterns differ by weight status (adjusting for socioeconomic status).

## Methods

Data are from the *Movement, Activity and Lifestyle – Tweens in Action* (MAL-TA) project, a cross-sectional study conducted between January and May 2012 with children in 54 schools in Malta [14]. A nationally representative sample of 1126 children was selected by the National Statistics Office (NSO) from a total population of 3890 (28.6%). This sample was stratified by regions of Malta and the neighbouring island of Gozo, type of school (495 from 35 state schools, 272 from 13 church schools, 107 from six independent schools) and gender (607 boys and 519 girls). One or two classes were chosen at random from each selected school and all children in each of these classes were invited to participate in the study. From the 1126 children invited, 901 children (80%) returned parental consent and the study was approved by the University of Malta research ethics committee.

### Children’s questionnaire

Participants completed a questionnaire with pre-coded answer categories to assess the time spent watching television (TV), using a computer for chatting, internet, emails or homework, and playing games on a computer or games console. Separate questions were asked for use on weekdays and weekends and categories included none, 1 minute to 30 minutes, 31 minutes – 1 hour, 1–2 hours, 2–3 hours, 3–4 hours, and more than 4 hours. Questions were adapted from the Health Behaviour in School Children study (HBSC) [12]. A previous study [15] reported satisfactory test-retest reliability for these questions. Data for TV viewing were recoded into less than 2 hours, and 2 hours or more (i.e. exceeding American Academy of Pediatrics guidelines [16]) while other ST was recoded into less than 1 hour and 1 hour or more. To facilitate comparison with data from the Health Behaviour of School Children study (HBSC) the data for ‘playing games on a computer and games console’ for over two hours was also analysed.

### Anthropometric measurements

Body height and weight were measured with the child in light clothing and without shoes. Height was measured using a SECA 213 Leicester Stadiometer (SECA, Hamburg, Germany) and recorded to the nearest 0.1 cm. Weight was recorded using a SECA 813 Digital Scale to the nearest 0.1 kg. Body mass index was calculated (kg/m^2^) and children were classified into normal weight, overweight and obese, using the age-related International Obesity Task Force criteria [17].

### Physical activity

Physical activity was assessed using Actigraph GT3X accelerometers (Actigraph, Pensacola, USA) set at 10 second epochs to capture children’s intermittent physical activity [18]. Participants were instructed to wear the accelerometer on an elastic belt over the right hip for five consecutive days including three weekdays and two weekend days. The accelerometer data were then processed using Kinesoft version 3.3.62 with a valid day based on at least 600 minutes of monitor wear. Periods of at least 60 minutes of continuous zeros were considered to be non-wear time and were removed [19]. Participants who had at least three days of valid data were included in this study [20]. The following accelerometer variables were then derived: total physical activity per day (in counts per minute), mean minutes of sedentary time per day, and mean minutes of moderate to vigorous physical activity (MVPA) per valid day. Time spent in these thresholds was classified based on the Evenson criteria of <100 CPM for sedentary and >2296 for MVPA [21]. In a rigorous study by Trost these cutpoints were found to provide the best classification accuracy [22].

Data were filtered by different time periods to enable an analysis of activity patterns during weekdays and weekend days. The morning period before schools was from 5.30 am to 8.29 am; the period at school from 8.30 am to 1.59 pm; the afternoon period from 2.00 pm to 6.59 pm and the evening time from 7.00 pm to 11.59 pm.

A search of the Biosis Citation Index, Derwent Innovations Index, Medline and Scielo Citation Index online databases was conducted in July 2013 using Web of Science. This identified articles that had cited Evenson’s 2008 [21] study. We manually screened the articles yielded from the search to identify articles which reported studies where a) accelerometry had been used to assess physical activity, b) comparable cutpoints for levels of intensity of activity had been used, and c) 10–11 year olds were the subjects of the study. While this was not a systematic review, it provided an opportunity to compare the results in the current study, with similar studies from other countries.

### Statistical analyses

Group means and standard deviations (SDs) were calculated for physical activity and sedentary time, and percentages for screen time. Independent sample t-tests were used to explore gender differences in activity and Chi-square tests were used for gender differences in screen time. Distributions were checked and as all variables approximated normality with relatively small deviations, parametric tests were used. Differences in physical activity by weight categories were analysed using analyses of covariance (ANCOVA) with the model adjusted for socioeconomic status. Chi-square tests were used to compare ST by weight status groups. To facilitate the development of targeted intervention strategies, separate analyses were performed for boys and girls, for weekdays and weekends, and for different periods of the day. Follow-up Bonferroni pairwise comparisons for significant main effects were calculated. All analyses were carried out using the IBM Statistical Package for Social Sciences (SPSS) version 21 and a p value of 0.05 was used for statistical significance.

## Results

From the 901 children who provided consent to participate in this study, 874 (97%) were present during the data collection period, that is 78% of the total sample invited (1126). All these children provided data for screen time, and of these, 811 (93% of the recruited children and 72% of the sample who had been invited) (412 boys and 399 girls) provided at least three days of valid accelerometer data, while 772 (88% of the recruited participants and 69% of those invited) provided data for weekend activity, and were included in the analyses. Children who provided accelerometer data that met inclusion criteria (n = 811) were not significantly different from those who failed to provide adequate data (n = 57) in gender, region or BMI category but those providing data had significantly higher socioeconomic status scores.

Using the International Obesity Task Force (IOTF) standards [17] 20.4% of the sample were overweight and 14.2% obese. A significantly greater percentage of boys than girls were overweight (24.2% v 16.4%) or obese (14.8% v 13.6%) (p=0.01).

### Physical activity

The means and standard deviations of boys and girls PA are presented separately for weekday and weekend days, in Table 1. The mean total PA for boys was 477.8 CPM (Confidence Interval [CI]–463.6-492.3) and for girls 385.0 CPM (CI–374.8-394.7) (p<0.001). Boys were engaged in 58.5 minutes of daily MVPA (CI–56.2-60.7), while girls’ MVPA was 42.2 minutes (CI–40.7-43.5) (p<0.001). Only a quarter of the children in this study (24.7%) met the daily recommendation of over 60 minutes of MVPA, and the percentage was higher for boys (39%) than girls (10%).

**Table 1:** Physical activity by gender for weekdays and weekends

**Weekdays**	**Boys (n = 412)**	**sd**	**95% Cl**	**Girls (n = 399)**	**sd**	**95% Cl**	**Total (n = 769)**	**sd**	**95% Cl**	**P value (t-test)**
Total activity (counts/min)	475.2	138.1	461.8-488.5	383.7	101.3	373.7-393.6	430.2	129.7	421.2-439.1	<0.001
Sedentary (min/day)	569.0	110.1	558.3-579.6	601.1	98.0	591.4-610.7	584.8	105.5	577.5-592.1	<0.001
MVPA (min/day)	59.0	22.5	56.8-61.2	43.3	14.9	41.8-44.7	51.3	20.7	49.8-52.7	<0.001
**MVPA (min/day)**										
5.30 am-8.29 am	5.6	3.7	5.2-5.9	4.9	3.1	4.6-5.2	5.2	3.4	5.0-5.5	0.005
8.30 am-1.59 pm	20.8	8.8	20.0-21.7	15.2	6.3	14.6-15.8	18.1	8.1	17.5-18.6	<0.001
2.00 pm-6.59 pm	24.3	13.8	23.0-25.6	17.2	8.6	16.3-18.0	20.8	12.1	20.0-21.6	<0.001
7.00 pm-11.59 pm	7.8	6.9	7.1-8.5	5.7	5.0	5.2-6.2	6.8	6.1	6.4-7.2	<0.001
**Weekends**	**Boys (n = 392)**			**Girls (n = 380)**			**Total (n = 774)**			
Total activity (counts/min)	477.9	202.0	457.8-498.0	388.4	161.5	372.1-404.7	433.9	188.5	420.5-447.2	<0.001
Sedentary (min/day)	550.1	170.4	533.2-567.1	556.9	158.3	540.1-572.9	553.5	164.5	541.8-565.1	0.567
MVPA (min/day)	57.2	32.0	54.0-60.4	40.1	21.4	38.0-42.3	48.8	28.6	46.8-50.8	<0.001
**MVPA (min/day)**										
5.30 am-8.29 am	1.7	2.8	1.4-1.9	1.0	1.8	0.8-1.2	1.3	2.3	1.2-1.5	<0.001
8.30 am-1.59 pm	25.3	18.2	23.5-27.1	16.7	11.3	15.6-17.9	21.1	15.8	20.0-22.2	<0.001
2.00 pm-6.59 pm	23.1	16.7	21.5-24.8	16.4	11.7	15.2-17.6	19.8	14.8	18.7-20.8	<0.001
7.00 pm-11.59 pm	6.0	6.0	5.4-6.6	5.1	5.6	4.6-5.7	5.6	5.8	5.2-6.0	0.032

Analysing the data by time-period, we found that boys were significantly more active than girls during all measured times. Weekday MVPA was highest during the period after school (2.00-6.59 pm), when boys were active for 24.3 minutes (CI–22.9-25.5), seven minutes more than girls (p<0.001). During the school day (8.30 am −1.59 pm), boys were engaged in 20.8 minutes of MVPA (CI–19.9-21.6), 5.6 minutes more than girls (p<0.001). On weekends, equal amounts of MVPA were obtained for the morning and afternoon periods for boys (25.3 and 23.1 minutes respectively), and for girls (16.7 and 16.4 minutes) (p<0.001).

### Screen time

The number and proportion of boys and girls in each ST category are presented separately for weekday and weekend days in Table 2. A high prevalence of over one hour of playing games on a computer or games console was reported by boys on weekdays (44.8%) and on weekends (51.6%) and these values are considerably higher than those reported for girls (28.1% and 35.0%) (p<0.001). Almost a third of boys (29.3%) watched over two hours of TV on weekends, compared to 20.6% of girls (p<0.001) and this prevalence is double that on weekdays.

**Table 2:** Screen time by gender for weekdays and weekends

**Screen time**	**Boys n**	**%**	**Girls n**	**%**	**Total n**	**%**	**P value (chi-square)**
**Weekdays**							
Watching TV	446		426		872		0.011
Less than 2 hours	375	84.1	383	89.9	758	86.9	
2 hours and more	71	15.9	43	10.1	114	13.1	
Using a computer for chatting, internet emails or homework	446		427		873		0.733
Less than 1 hour	316	70.9	307	71.9	623	71.4	
1 hour and more	130	29.1	120	28.1	250	28.6	
Playing games on a computer or games console	446		427		873		<0.001
Less than 1 hour	246	55.2	307	71.9	553	63.3	
1 hour and more	200	44.8	120	28.1	320	36.7	
**Weekends**							
Watching TV	444		422		866		0.003
Less than 2 hours	314	70.7	335	79.4	649	74.9	
2 hours and more	130	29.3	87	20.6	217	25.1	
Using a computer for chatting, internet emails or homework	447		427		874		0.873
Less than 1 hour	315	70.5	303	71.0	618	70.7	
1 hour and more	132	29.5	124	29.0	256	29.3	
Playing games on a computer or games console	446		426		872		<0.001
Less than 1 hour	216	48.4	277	65.0	493	56.5	
1 hour and more	230	51.6	149	35.0	379	43.5	

### Comparison with other countries

Table 3 provides a comparison of the results of the current study and previously published studies. Using the same Evenson cut points [21], mean MVPA of Maltese boys and girls were marginally higher than those of children in Philadelphia, USA [23], while compared to data from Canberra, Australia [24], mean MVPA of Maltese boys and girls were considerably higher. In contrast, means were much lower than those in the PEACH study carried out in Bristol, England [25]. Comparing median MVPA values to those of children in a longitudinal study of the National Institute of Child Health and Human Development (NICHD) in ten geographical locations in the USA, Maltese children were again more active [26]. The percentage of Maltese children meeting recommendations for PA (60 minutes a day) was slightly lower than that of children in USA, while compared to Australian data, percentages were higher for Maltese boys and lower for Maltese girls.

**Table 3:** **A comparison of objectively assessed MVPA using Evenson **[21]** cut-offs and screen viewing in other countries**

**Study**	**Age**	**Gender**	**n**	**Mean MVPA (min)**	**Median MVPA (min)**	**Meeting recommendation (%)**
Present study 2012 - Malta	10.8	Boys	412	58.1	54.8	39
	10.7	Girls	399	41.7	39.7	10
Cooper et al.,2012 [25] - England	11.0	Boys	250	70.1		NR^*^
		Girls	315	56.0		
Trost et al.,2013 [23] - USA	10-11	Boys	201	57.5		41.5
		Girls	269	35.5		11.5
Mitchelle et al.,2013 - USA	11.0	Boys	369		46.7	NR
		Girls	382		32.4	NR
Telford et al.,2013 [24] – Australia	11.1	Boys	282	43.0		33
		Girls	266	31.0		18
^*^NR - Not reported						
**Percentage spending 2 hours or more of weekday screen viewing (all 11 years olds)**						
			**Watching TV**		**Playing games on a computer and games console**	
			**Boys%**	**Girls%**	**Girls%**	**Boys%**
Present study (Malta)			16	10	12	25
HBSC* Average			58	54	22	40
Highest HBSC						
Ukraine			69	71		
Romania					43	57
Lowest HBSC						
Switzerland			29	24	8	16
Canada HBSC			64	56	25	45
USA HBSC			56	50	17	31

^*^HBSC - Health Behaviour in School-Aged Children study (Currie et al. [3]).

Comparing the percentages of children exceeding two hours of screen time on weekdays in this study to other countries in Europe, Canada and USA, using the Health Behaviour in School-aged children (HBSC) study [3] as a comparator, we found substantially lower rates in Maltese children. TV watching percentages for Maltese children were lower than children in all other countries, while for playing games on computers and games consoles, they were only higher than the lowest HBSC values observed in Switzerland (and boys in Luxembourg).

### Physical activity and screen time by weight status

Table 4 provides a presentation of PA by weight status. For weekdays the overall pattern is that MVPA and total PA differ by weight group. Follow-up tests indicated differences between the normal weight and obese and for boys on a weekday there were also differences between the overweight and obese groups. There were similar patterns when the analyses were repeated for the separate time periods. In all instances PA levels were lower in the obese group than the normal weight group. There were significant differences between the TV viewing times of boys in different weight categories on weekends only. Almost half of obese boys (45.5%) watched over two hours of TV compared to 30.8% of overweight and 24.7% of normal weight boys (p=0.004) (see Table 5). Obese boys (45.5%) were also more engaged in using a computer for chatting, emails or homework than overweight (28.0%) and normal weight boys (25.6%) (p=0.006), however this was significant only on weekdays. No significant differences were observed in the screen time of girls in different weight categories. In contrast, objectively measured overall sedentary time of overweight boys was significantly lower than that of normal weight boys both on weekdays and on weekends (see Table 4). Differences in girls were not statistically significant.

**Table 4:** MVPA during different times of the day by weight status (model adjusted for socioeconomic status)

	**Normal weight**	**sd**	**95% Cl**	**Overweight**	**sd**	**95% Cl**	**Obese**	**sd**	**95% Cl**	**Total**	**sd**	**95% Cl**	**P value (ANCOVA)**	**Post-hoc (Bonferroni)**
**Boys**														
**Weekdays**	(n = 254)			(n = 99)			(n = 59)			(n = 412)				
MVPA (min/day)	61.2	22.2	58.6-64.0	59.2	23.8	54.4-64.0	49.1	18.8	44.6-54.3	59.0	22.5	56.9-61.2	0.001	B, C*
Sedentary (min/day)	582.2	112.9	568.5-596.0	544.5	106.9	524.5-565.0	552.7	94.3	529.6-577.8	569.0	110.1	558.2-579.1	0.009	A
Total activity (counts/min)	481.4	135.1	464.7-497.4	487.3	149.8	458.5-519.0	428.3	122.4	398.0-462.1	475.2	138.1	462.4-488.8	0.015	B, C
5.30 am-8.29 am (counts/min)	477.6	244.8	447.4-508.5	495.3	206.6	452.9-538.9	480.8	194.8	428.5-530.7	482.3	229.1	459.9-504.8	0.846	
8.30 am-1.59 pm (counts/min)	465.0	141.6	447.8-481.7	468.3	167.2	436.1-502.5	442.5	164.7	403.1-482.5	462.6	151.3	448.6-477.6	0.466	
2.00 pm-6.59 pm (counts/min)	581.2	250.8	550.7-610.8	581.8	235.3	534.4-631.3	470.4	173.7	425.5-518.4	565.5	240.2	540.5-588.6	0.004	B, C
7.00 pm-11.59 pm (counts/min)	391.9	244.4	362.1-422.0	382.7	211.6	342.8-426.1	341.6	151.2	305.5-377.4	382.4	225.7	360.5-404.9	0.301	
**Weekends**	(n = 241)			(n = 93)			(n = 57)			(n = 391)				
MVPA (min/day)	60.4	32.6	56.6-64.4	55.2	30.5	49.4-61.5	46.9	29.7	39.6-55.3	57.2	32.0	54.1-60.2	0.012	C
Sedentary (min/day)	572.3	185.8	549.9-597.2	512.6	133.1	487.0-538.4	518.0	140.8	484.9-554.1	550.1	170.4	534.0-567.9	0.005	A
Total activity (counts/min)	488.3	209.4	462.3-515.9	476.6	189.2	439.5-516.1	436.2	188.0	387.8-487.2	477.9	202.0	458.4-499.4	0.206	
5.30 am-8.29 am (counts/min)	382.6	522.0	310.6-468.4	465.7	464.0	365.4-581.3	344.2	281.5	266.8-427.9	396.4	479.5	341.8-455.4	0.316	
8.30 am-1.59 pm (counts/min)	567.7	289.1	533.0-605.4	617.9	341.8	549.5-684.4	484.4	231.4	430.3-541.5	567.5	297.1	538.5-598.5	0.028	B
2.00 pm-6.59 pm (counts/min)	578.9	324.9	542.2-625.2	495.8	224.2	449.3-542.9	486.5	269.0	422.0-562.5	545.6	298.1	517.8-575.8	0.017	A
7.00 pm-11.59 pm (counts/min)	329.0	193.7	301.6-356.6	306.5	151.9	273.7-337.6	373.2	255.0	312.1-448.8	330.0	195.4	309.3-350.3	0.168	
**Girls**														
**Weekdays**	(n = 280)			(n = 64)			(n = 54)			(n = 398)				
MVPA (min/day)	44.1	15.7	42.4-46.1	43.7	13.5	40.3-47.0	38.3	11.5	35.2-41.5	43.3	14.9	41.9-44.8	0.032	C
Sedentary (min/day)	603.4	96.6	592.2-614.7	583.6	75.7	565.3-602.3	610.3	125.0	580.7-644.8	601.2	98.1	591.3-610.2	0.21	
Total activity (counts/min)	387.8	103.4	375.5-400.0	393.2	95.5	369.5-418.5	351.3	93.1	327.3-376.8	383.7	101.4	374.4-393.4	0.034	C
5.30 am-8.29 am (counts/min)	430.7	187.6	409.6-454.0	423.9	177.1	383.0-473.3	436.6	164.3	391.8-481.2	430.4	182.5	412.3-448.9	0.956	
8.30 am-1.59 pm (counts/min)	365.5	122.3	352.3-379.8	354.8	105.4	331.6-381.6	345.3	114.9	316.2-377.5	361.0	118.7	350.1-372.3	0.466	
2.00 pm-6.59 pm (counts/min)	453.5	170.5	432.9-474.1	467.4	166.0	429.8-504.3	386.6	128.1	352.8-422.6	446.6	166.1	431.1-462.7	0.012	B, C
7.00 pm-11.59 pm (counts/min)	335.1	194.6	312.5-357.9	327.2	163.1	289.2-369.9	288.8	128.4	255.1-321.5	327.5	182.4	309.3-345.4	0.234	
**Weekends**	(n = 267)			(n = 61)			(n = 50)			(n = 378)				
MVPA (min/day)	41.8	22.8	39.0-44.5	38.2	17.1	34.3-42.4	33.7	17.4	29.1-38.6	40.1	21.4	38.0-42.4	0.049	C
Sedentary (min/day)	558.3	155.6	540.1-577.4	549.3	158.3	510.4-593.7	556.0	175.1	510.4-604.6	556.6	158.4	540.7-572.9	0.921	
Total activity (counts/min)	397.1	166.5	376.8-417.3	386.7	154.4	348.8-428.2	345.7	137.5	309.4-382.7	388.6	161.6	372.1-405.4	0.143	
5.30 am-8.29 am (counts/min)	385.2	549.9	312.6-467.1	279.2	257.1	194.2-369.6	474.2	601.0	322.4-720.1	381.8	527.4	321.2-450.6	0.299	
8.30 am-1.59 pm (counts/min)	444.0	209.6	418.6-469.1	418.5	191.5	372.9-466.7	373.9	162.7	331.7-419.2	430.7	202.1	410.2-450.9	0.084	
2.00 pm-6.59 pm (counts/min)	457.1	259.0	427.5-491.4	426.4	267.1	367.6-496.6	366.6	188.3	317.9-418.1	440.3	253.5	414.9-468.3	0.073	
7.00 pm-11.59 pm (counts/min)	306.9	212.2	281.0-332.7	318.8	178.1	273.6-366.7	279.8	147.1	236.4-324.2	305.2	199.1	284.7-328.2	0.563	

*A = Normal weight vs overweight p < .05.

B = Overweight vs obese p < .05.

C = Normal weight vs obese p < .05.

**Table 5:** Percentages for screen time by gender and weight status

**Watching TV**							**Using a computer for chatting, internet emails or homework**					**Playing games on a computer or games console**				
	**Normal weight**	**Overweight**	**Obese**	**Total**	**P value (chi-square)**		**Normal weight**	**Overweight**	**Obese**	**Total**	**P value (chi-square)**	**Normal weight**	**Overweight**	**Obese**	**Total**	**P value (chi-square)**
**Boys**																
**Weekdays**						**Weekdays**										
n	272	108	66	375	0.978	n	273	107	66	446	0.006	272	108	66	446	0.842
<2 hours -%	83.8	84.3	84.8	84.1		<1 hour -%	74.4	72.0	54.5	70.9		56.3	53.7	53.0	55.2	
≥2 hours -%	16.2	15.7	15.2	15.9		≥1 hour -%	25.6	28.0	45.5	29.1		43.8	46.3	47.0	44.8	
**Weekends**						**Weekends**										
n	271	107	66	444	0.004	n	273	108	66	447	0.415	272	108	66	446	0.266
<2 hours -%	75.3	69.2	54.5	70.7		<1 hour -%	71.4	72.2	63.6	70.5		51.5	44.4	42.4	48.4	
≥2 hours -%	24.7	30.8	45.5	29.3		≥1 hour -%	28.6	27.8	36.4	29.5		48.5	55.6	57.6	51.6	
**Girls**																
**Weekdays**						**Weekdays**										
n	298	69	58	425	0.662	n	298	70	58	426	0.221	298	70	58	426	0.142
<2 hours -%	90.6	87.0	89.7	89.9		<1 hour -%	74.2	68.6	63.8	71.8		74.5	70	62.1	72.1	
≥2 hours -%	9.4	13.0	10.3	10.1		≥1 hour -%	25.8	31.4	36.2	28.2		25.5	30.0	37.9	27.9	
**Weekends**						**Weekends**										
n	294	69	58	421	0.148	n	298	70	58	426	0.346	298	70	57	425	0.277
<2 hours -%	81.0	71.0	82.8	79.6		<1 hour -%	72.8	64.3	69.0	70.9		67.1	64.3	56.1	65.2	
≥2 hours -%	19.0	29.0	17.2	20.4		≥1 hour -%	27.2	35.7	31.0	29.1		32.9	35.7	43.9	34.8	

## Discussion

In this study we found that 39% of boys and 10% of girls met PA guidelines. Additionally, 29.3% of boys and 20.6% of girls exceeded the American Academy of Pediatrics (AAP) [27] guideline by watching more than 2 hours of TV per day on a weekend, and these percentages were double those observed for weekdays. Maltese children were found to be more active than children in the USA and Australia, were fewer active than English children, and spent fewer hours in front of a screen than children in other countries. As published elsewhere [14], a high percentage of children in this study were found to be overweight or obese, particularly boys, making them amongst the fattest in the world. The data from this study therefore indicate that although Maltese children are quite active compared to children in other countries, they are still more likely to be obese. Additionally, Maltese boys were more active than girls throughout the week and also less sedentary on weekdays, however boys are more overweight or obese. As concluded in a recent review by Wilks et al. [28] these international comparisons suggests that physical inactivity may not be the major contributor to the development of obesity in **all** children.

However, when categorised as BMI groups, the data reported here indicate that obese children engaged in a significantly lower total volume of activity and MVPA than overweight and normal weight children. Overweight and obese children also spent more hours in front of a screen compared to normal weight children. These results contribute to the debate on the relationship between PA and obesity, recently summarised in a review [6] which has reported mixed associations. The data therefore suggest that there are associations between PA/ST and obesity but the direction of causality of these associations cannot be delineated from this cross-sectional dataset. Although lower activity levels and greater time spent sedentary might contribute to weight gain in children, it is equally plausible that overweight and obese children become less active. Extra weight requires more exertion for physical tasks, and being overweight during activity may attract negative reactions from others, and cause discouragement. Moreover, one cannot determine fully that obesity is linked to increased screen time, or that it is due to behaviours such as increased snacking on energy dense food during screen time, particularly TV viewing.

This study found contrasting results for self-reported screen time and objectively measured sedentary behaviour. This confirms the recent findings of Verloigne et al. [7] that self-reported TV and computer time do not reflect total sedentary time in children. Accelerometry does not tell us what children are doing, therefore we need more studies that investigate other sedentary behaviours. Although screen time might be taking a good amount of children’s time, children are also spending a considerable amount of time in other sedentary activities at school and in extra study at home.

In this study we found that a low percentage of children, particularly girls meet daily PA guidelines. As found in other studies, girls were less active than boys throughout the week [29,30] stressing the need to provide them with more opportunities for physical activity, while aiming to raise the levels of activity of all the children. Verloigne et al. attributed these gender differences to the social context at particular time-periods [31]. Boys might be dominating playgrounds at school, and girls prefer to socialise during breaks [32], while after school, parents might be considering neighbourhoods to be safe for boys, but not for girls [33].

In contrast to previous research [29,30,34], the overall patterns of activity on weekends were not different from weekdays. Although children spend almost half their waking time at school, we found that they were only engaged in a quarter of the recommended hour of MVPA. Schools have been described as the providers of the best opportunity for a population-based approach to increasing physical activity [35]. Therefore adopting a whole school approach to increase physical activity through the development and implementation of physical activity policies is a priority. Girls should also be encouraged to be as active as boys and activities should be made appealing to them. Thirty minutes of daily MVPA at school is an achievable target for all children. This could be partly achieved through daily physical education lessons where at least 50% of the time is spent in MVPA, and this will result in long term benefits through the development of physical literacy which is key for lifelong physical activity. Introducing active breaks in class could also increase activity levels while having potential to stimulate improved academic performance [36]. As recommended by several researchers [37] more activity can be done during recess, when children can engage in up to twenty minutes of MVPA.

In the current dataset, the after school period provided only a few more minutes of activity than during school time. Half an hour of MVPA during this period [38,39], would result in children accumulating a total of one hour of daily MVPA. After-school sport programmes specifically targeting girls may provide one solution, together with more opportunities for unstructured play and access to open areas in the community. Furthermore, as suggested by Jago et al. [40], encouraging girls to support their friends’ activity is important in order to help them sustain PA levels during periods of transition from primary to secondary schools. More opportunities for girls to be active on weekends, when they are about 15 minutes less active than boys, could be considered, although this would require strategies to engage parental support. Finally, another time-period when activity was very low is the period before school, when children can be encouraged to walk to school or be active while they wait outside or inside the school premises.

Weight category differences during periods after school hours might suggest the need for specific interventions to increase PA after school among overweight children. Although differences in total activity by weight status during school hours were not significant, the school still remains the place where activity for obese children can be increased, where they can gain the skills and confidence for being active outside school hours and throughout their life. Establishing a link between schools and clubs to encourage children of all abilities and sizes to become active would help. Holding such clubs on the school premises might provide a more reassuring environment for obese children, while providing more opportunities for unstructured play and physical activity in the community is also needed.

### Strengths and limitations

The strength of this study is its use of objective measurement of physical activity and weight status with a nationally representative sample of Maltese 10–11 year olds. A large percentage of the sample selected (80%) accepted to participate in this study, however a limitation might be that we do not know the weight status of those who did not provide consent. It is usually parents of obese children who refuse consent in studies on obesity [41]. Therefore if this is the case, the sample may be under representative of overweight and obese children. Although accelerometry has been used to provide measures of intensity and duration of physical activity, current data cannot describe what the participants are doing while they are sedentary or active and cannot assess some activities like swimming and cycling. This study has used Evenson’s cutpoints and other higher accelerometer cutpoints would have produced lower prevalences of children meeting PA guidelines. A further limitation was that we were not able to calculate energy expenditure and the results provide simple estimates of movement. This underestimates the energy expenditure during activity of children who are heavier. Screen time was self-reported, and considering that children engage in many different types of screen time, the measure is likely to lack precision and accuracy. It is important to recognise that other factors including socio-economic position could also have affected the associations found in this study. We relied on evidence of the reliability and validity of sections of the questionnaire from other studies and cannot confirm how robust the measures were in our sample. Children were classified into different weight categories using the IOTF standards, and although these standards are used in many studies for international comparison, another study in Malta [14] has shown that these standards produce lower prevalence of overweight and obesity compared to WHO standards.

## Conclusions

A low percentage of Maltese 10–11 year olds (39% of boys, 10% of girls), particularly girls, reached the recommended levels of daily MVPA and spent large amounts of time engaged in screen time. Obese children were less active than non-obese children, reaching significance at particular key periods during the week and also had higher levels of screen time. As children spend most of their waking time at school and that activity during this time is less than one third of the daily requirements, aiming to increase MVPA at school for all Maltese children is likely to be important and strategies to promote MVPA during this time periods may be helpful.

## Competing interests

The authors declare that they have no competing interests.

## Authors’ contributions

This paper is based on research conducted by AD as part of his PhD, supervised by RJ and KRF. AD collected the data, performed the statistical analysis, and drafted the manuscript. All authors have contributed to the conception, design of the study and in writing the paper. All authors approved the final manuscript.

## Pre-publication history

The pre-publication history for this paper can be accessed here:

http://www.biomedcentral.com/1471-2458/14/664/prepub
